# Recreational Screen-Time Among Chinese Adolescents: A Cross-Sectional Study

**DOI:** 10.2188/jea.JE20140006

**Published:** 2014-09-05

**Authors:** Xiao-Xiao Jiang, Louise L. Hardy, Ding Ding, Louise A. Baur, Hui-Jing Shi

**Affiliations:** 1 School of Public Health, Fudan University/Key Laboratory of Public Health Safety, Chinese Ministry of Education, Shanghai, China; 2 Prevention Research Collaboration, School of Public Health, University of Sydney, NSW, Australia

**Keywords:** screen time, adolescents, China, urban

## Abstract

**Background:**

Rapid urbanization in China has led to a proliferation of electronic entertainment media among youth. Prolonged screen time (ST; includes watching television and playing on computers, video game consoles, or mobile phones) is linked to poor health profiles. The aim of this study was to report recreational ST behaviors and ST correlates among Chinese adolescents living in two regions with different degrees of urbanization.

**Methods:**

A cross-sectional, school-based survey (*n* = 3461 adolescents; aged 12–14 years old) living in inner-city Shanghai and a peri-urban region of Hangzhou. Students completed a questionnaire including family characteristics, daily ST, and information on family environment related to screen use. Recreational ST was categorized into two groups according to recommendations by the American Academy of Pediatrics (< or ≥2 h/day). Parents reported their own ST and also reported educational attainment as a proxy for socioeconomic status.

**Results:**

ST was higher among boys than girls and on weekends than weekdays. Peri-urban girls were more likely to exceed 2 h/day ST compared to inner-city girls on weekends. Having a father with no university degree, mother’s TV viewing ≥2 h/day, no ST rules at home, and eating meals in front of the TV were associated with higher ST on both weekdays and weekends, and regional differences were found for weekend ST.

**Conclusions:**

TV viewing and playing on the computer were the most prevalent ST behaviors among Chinese adolescents. Mobile phone playing was less prevalent but persistent throughout the week. More population-level surveillance and research is needed to monitor the trends in ST behaviors and to better understand the characteristics of those who are at risk.

## INTRODUCTION

China is the world’s second largest economy, after the United States.^1^ The World Bank has deemed the standard of living in large Chinese cities as “high middle income,” and there is accumulating evidence of changes in weight-related behaviors, such as diet physical activities, and sedentary behaviors in young Chinese.^2^^,^^3^ Furthermore, rapid globalization and urbanization have created greater accessibility to screen devices, including television (TV), computers, and video game consoles, among Chinese adolescents.^4^

Prolonged screen time (ST) has been associated with adverse metabolic and psychosocial health indices in adolescents and children.^5^^,^^6^ Among young people, the usage of screen devices increases with age and peaks in adolescence. However, the evidence base for ST correlates mainly comes from studies conducted in Western countries.^7^^,^^8^ Epidemiological data on the prevalence, distribution, and correlates of ST among non-Western youth may differ from those among Western youth.

While the research on ST among Chinese children is very limited, there is some evidence that patterns of ST behaviors among Chinese youth vary by levels of urbanization, with urban adolescent boys showing the largest increase between 1997 and 2006 compared with urban girls and rural counterparts.^9^ However, given the rapid social and economic transitions in China and the rising popularity of mobile screen devices (eg, smart phones), more recent data are required to determine current ST patterns and whether there are differences between regions in different stages of urbanization. In the context of metropolitan China, ‘urban’ refers to both inner-city and peri-urban areas. Peri-urbanization refers to the process of dispersal of urban growth toward the rural surroundings of cities (urban sprawl), which creates landscapes that are characterized by both urban and rural social and economic activities.^10^^,^^11^ We hypothesized that, compared with children from peri-urban areas, children from more urbanized areas will be more likely to engage in prolonged ST behavior and have greater access to screen devices such as mobile phones.

The objective of this study was to report more recent epidemiology of recreational ST behavior (including mobile phones and ‘tablets’) outside of school hours on weekdays and weekends than has been previously reported, and to examine the correlates of the home screen environment with prolonged ST in adolescents residing in two Chinese cities with different degrees of urbanization.

## METHODS

### Sampling and procedures

A cross-sectional, school-based survey was conducted among students in grades 7 and 8 (12–14 years old) living in inner-city Shanghai and Yuhang, the peri-urban area of Hangzhou, which are both located in one of China’s most economically active regions. Hangzhou is an affluent city in eastern China, 180 km from Shanghai. The peri-urban area in Hangzhou is less urbanized than Shanghai, with some farming activity coexisting with other industries. Thus, the selection of this area enabled us to capture a variety of urban environments where Chinese adolescents might reside.

Data were collected between October and November 2011 by trained field staff responsible for administering questionnaires and answering queries from participants. Written informed consent was a requirement for participation in the survey. Participants were sampled from the region where over 99% of the population was of Han ethnicity.^12^ The study was mainly funded by the Shanghai Municipal Committee of Education and approved by the Ethics Review Board of Fudan University (IRB # 2011-12-0321).

A multi-stage cluster sampling design was used to select districts, schools, and classes. All students in selected classes were invited to participate in the study. Based on sample size calculation for clustered design (intra-class correlation was conservatively set as 0.05, design effect = 3), 3672 participants were needed, with an average of 40 students per class. The first stage involved the random selection of four of the nine districts in inner-city Shanghai and one of the two peri-urban areas in Hangzhou. The second stage involved random selection of schools (three middle schools from each district) and the third stage involved the selection of classes (115 classes from 15 schools).

### Measures

Students reported sex and school grade. Body weight (kg) and height (m) were measured by clinical staff in the spring of 2011, and body mass index (BMI) was calculated as weight/height^2^. BMI was classified into three categories (healthy, overweight, and obese) according to the International Obesity Taskforce age- and sex-specific BMI cut-points for the Asian pediatric population.^13^ Recreational ST was reported separately for an average week and weekend day outside of school hours and included TV viewing, playing on the computer (desktop, laptop, and tablet devices), playing video game consoles, and playing on their mobile phones. Playing on the computer was defined as playing games, surfing the Internet, and watching movies. Playing on the mobile phone was defined as playing games and surfing the Internet. Students reported the time (hours and minutes) separately for each screen activity, and total ST was calculated. Ten-day test-retest reliability of the questionnaire was satisfactory (intra-class correlation coefficients: 0.78 for weekday and 0.82 for weekend). For an average weekday, students were asked to recall ST before school, on the way to school, on the way home from school, and after school. For weekends, students were asked to recall the time for an average weekend day.

Students also reported information on the family screen environment including rules (“Do your parents have rules regarding your screen-time behaviors?” [Yes/No]); and eating while watching TV (“Does your family usually have meals while watching TV?” [Yes/No]). Separate questions were asked on whether there was a TV, computer, or video game device in their bedroom (Yes/No), and whether they owned a personal mobile phone (Yes/No). The number of screen devices in the bedroom was summed according to responses to these four questions, and the proportion within each sub-group having 0 to 3 devices was calculated. One parent or guardian of each student also completed a questionnaire including information on their educational level (whether or not they had a university degree), the number of children currently living at home, and the average daily time each parent spent watching TV and using a computer in the preceding 7 days. The student questionnaires were completed during one class session, and the parental questionnaires were completed by the parents/guardians at home and brought back to school by the students.

### Data analysis

Data were analyzed in March 2013 using IBM SPSS (version 19 for Windows, Chicago, IL, USA). An SPSS Complex Samples package was used to account for the clustered data structure, which is equivalent to conducting mixed-model analyses specifying class as a random-effect variable (this was confirmed by rerunning a sample of analyses by using generalized linear mixed models in SPSS). Statistical significance was set at *P* < 0.05. The distributions of all ST variables were zero-inflated. Therefore, we presented the proportion of students engaging in each ST behavior (ie greater than 0 minutes/day) and the mean and standard deviation of ST time for those who reported a non-zero value for each ST behavior.

ST was summed (h/day), and the proportion of students meeting the ST recommendation (<2 h/day)^14^ was calculated for weekdays and weekend days, stratified by sex and region. Differences between groups were examined by independent *t*-test and Chi-square test. Associations between potential correlates (eg, socio-demographic characteristics, home screen environments) and meeting the ST recommendation (<2 h/day) were assessed using logistic regression (all variables were mutually adjusted in the models). Multicollinearity was assessed (variance inflation factors [VIF] 1.02 to 1.37), but there was no evidence of multicollinearity. For multivariate analysis, the list-wise deletion method was used for handling missing data, and the students were excluded from analyses if any single value was missing. The difference in demographic characteristics between the excluded students and the remaining students was compared by *t*-test or Chi-square test. Due to missing data on covariates, the numbers of participants excluded from the analyses of weekday and weekend screen time were 558 and 511, respectively. Analyses revealed no significant differences between excluded and non-excluded adolescents in terms of basic characteristics (gender, age, and parents’ education) or the dependent variable (proportion of students exceeding 2 h/day ST).

## RESULTS

### Sample characteristics

In total, 3461 adolescents participated in the survey and had completed parent reports (inner-city Shanghai = 2332, response rate 93.3%; peri-urban Hangzhou = 1129, response rate 90.5%). The characteristics of the adolescents are presented in Table 1, stratified by region and sex. The mean age was 13.2 (standard deviation, 0.7) years, and 49.9% of the sample were boys. Overall, parents of inner-city adolescents were more likely to have tertiary education than peri-urban parents (*P* < 0.001), and more than a quarter (25.7%) of the adolescents were living with a sibling, with the prevalence higher among girls than boys (*P* < 0.001).

**Table 1.** Sample characteristics by region and sex (*n* = 3461)

	Shanghai (Inner city)		Yuhang (Peri-urban)	
	
	Boys	Girls	Boys	Girls
Sample size, *n* (%)	1170 (50.2)	1162 (49.8)	557 (49.3)	572 (50.7)
Age in years, mean (SD)	13.2 (0.7)^a,b^	13.1 (0.7)^b^	13.2 (0.6)	13.3 (0.6)
Overweight or obese (%)	47.7^a,b^	31.6^b^	19.3^a^	8.8
*Parents with university degree (%)*				
Father	23.5^b^	22.4^b^	8.3	11.6
Mother	18.0^b^	18.5^b^	4.5	7.2
Lives with a sibling (%)	24.2^a^	28.3	21.8^a^	27.3
*Parents’ screen time ≥2 h/day (%)*				
Father	78.2	81.7	77.0	81.5
Mother	76.4	79.0	77.8	77.4
*Family screen environment (%)*				
No family rule on ST	49.9	50.9	46.3	51.9
Family eats meals while watching TV	43.6^b^	41.2^b^	25.1	24.8
TV in bedroom	37.3^a,b^	32.2	31.1	29.2
Computers in bedroom	37.7^a,b^	43.1^b^	21.4	22.9
E-games devices in bedroom	21.0^a,b^	14.8^b^	29.6^a^	10.3
Own mobile phone	66.0^a,b^	80.9^b^	46.9^a^	62.1
Number of screen devices in the adolescent’s bedroom (%)				
0	40.9	42.2^b^	43.5^a^	54.2
1	30.7	31.6	35.1	31.3
2	19.4	20.2^b^	17.3^a^	12.4
3	8.9^a,b^	6.0^b^	4.1	2.1

SD, standard deviation; ST, screen time; TV, television.

^a^*P* < 0.05 between boys and girls of same region (independent *t*-test for continuous variables and Chi-square test for categorical variables).

^b^*P* < 0.05 between regions of same sex.

### Family screen-time environment

Overall, more than three quarters of the parents reported ≥2 h/day ST, and 50% of adolescents reported that there were no family rules for ST. Inner-city adolescents were more likely to report having meals while watching TV than peri-urban adolescents (42.4% vs 25.0%; *P* < 0.001) and rates of having a computer in the bedroom compared with having a TV in the bedroom (40.4% vs 34.7%; *P* < 0.001). In contrast, peri-urban adolescents were more likely to have a TV in their bedroom than a computer (30.1% vs 22.1%; *P* < 0.001). Mobile phone ownership was higher among inner-city adolescents than among their peri-urban counterparts. Approximately 60% of inner-city adolescents had one or more screen devices in their bedrooms, and a higher proportion of inner-city adolescents had three screen devices in their bedroom than their peri-urban counterparts.

### Daily ST by device

Table 2 shows the proportion of adolescents who reported any recreational ST (ie >0 minutes) and the mean daily minutes spent on each screen device for all adolescents who reported non-zero ST. Overall, the respective mean daily weekday and weekend recreational STs were 84.3 and 240.7 minutes for boys and 71.2 and 202.5 minutes for girls. The proportions of adolescents who reported ≥2 h/day of total ST on weekdays and weekends were 15.8% and 72.6%, respectively. The most prevalent recreational ST behavior was watching TV, and the least was playing video game consoles for both weekdays and weekends. The largest ST difference between weekdays and weekends was in computer playing, with the prevalence being three times higher on weekends than on weekdays. No significant difference (*P* = 0.22) was found for the prevalence of mobile phone playing between weekdays and weekends.

**Table 2.** Screen-time by region, sex, and type of day

	Shanghai (Inner city)		*P*	Yuhang (Peri-urban)		*P*	*P*_boy_	*P*_girl_
	
	Boys (*n* = 1170)	Girls (*n* = 1162)		Boys (*n* = 572)	Girls (*n* = 557)			
**Weekday**								
TV viewing (minutes/day)								
>0 minutes (%)	66.4	51.2	<0.001	56.6	46.4	0.003	<0.001	0.09
Mean (SD)^a^	54.0 (43.2)	49.2 (40.7)	0.053	63.2 (45.0)	51.9 (38.8)	0.004	0.006	0.40
Computer playing (minutes/day)								
>0 minutes (%)	25.1	26.0	0.65	16.5	15.5	0.72	<0.001	<0.001
Mean (SD)^a^	68.5 (63.0)	50.9 (44.1)	<0.001	56.7 (40.9)	42.3 (30.8)	0.02	0.06	0.12
Video game playing (minutes/day)								
>0 minutes (%)	7.5	2.9	<0.001	3.2	0.7	0.006	0.002	0.004
Mean (SD)^a^	53.1 (59.2)	30.4 (28.0)	0.048	39.5 (34.7)	50.0 (26.5)	0.63	0.41	0.26
Mobile phone playing								
>0 minutes (%)	26.0	40.8	<0.001	19.2	27.2	0.006	0.006	<0.001
Mean (SD)^a^	43.7 (48.0)	36.5 (34.5)	0.04	37.5 (30.4)	40.9 (37.3)	0.48	0.26	0.22
Total screen time mean (SD)^a^	86.1 (103.5)	72.1 (71.9)	0.002	79.6 (58.0)	68.8 (54.8)	0.02	0.02	0.20
Screen time exceeding 2 h/day % (*n*)	18.9 (187)	13.0 (133)	<0.001	19.1 (83)	12.2 (56)	0.005	0.94	0.74
**Weekend**								
TV viewing (minutes/day)								
>0 minutes (%)	78.7	75.8	0.12	85.0	85.2	—	0.002	<0.001
Mean (SD)^a^	127.3 (90.0)	112.2 (82.3)	<0.001	134.6 (85.9)	127.3 (79.5)	0.18	0.15	0.001
Computer playing (minutes/day)								
>0 minutes (%)	71.3	68.5	0.17	65.2	61.1	0.16	0.01	0.003
Mean (SD)^a^	136.4 (100.9)	109.2 (81.2)	<0.001	131.3 (90.8)	108.0 (74.6)	<0.001	0.41	0.81
Video game playing (minutes/day)								
>0 minutes (%)	14.0	4.9	<0.001	7.2	1.8	<0.001	<0.001	0.001
Mean (SD)^a^	81.3 (67.0)	61.2 (55.8)	0.05	91.4 (58.9)	49.5 (26.9)	0.03	0.39	0.52
Mobile phone playing								
>0 minutes (%)	26.9	38.9	<0.001	21.4	27.3	0.03	0.02	<0.001
Mean (SD)^a^	75.6 (89.4)	61.1 (62.8)	0.02	69.5 (68.0)	65.3 (57.8)	0.58	0.50	0.47
Total screen time mean (SD)^a^	245.4 (185.6)	201.2 (152.7)	<0.001	231.6 (149.3)	205.1 (130.8)	0.002	0.04	0.06
Screen time exceed 2 h/day % (*n*)	75.7 (841)	66.2 (733)	<0.001	79.3 (440)	72.5 (412)	0.01	0.11	0.008

SD, standard deviation; TV, television.

*P*: significance test between boys and girls.

*P*_boy_: significance test between regions among boys.

*P*_girl_: significance test between regions among girls.

^a^All means were calculated for subjects who reported non-zero time. Independent *t*-test and Chi-square test were used to examine the difference.

On weekdays, total recreational ST was higher among inner-city adolescents than peri-urban adolescents. However, significant regional differences in exceeding 2 h/day of ST were only observed among girls on weekends (*P* = 0.008). Inner-city boys spent more time playing on a computer but less time watching TV than peri-urban boys, and peri-urban girls reported watching more TV than inner-city girls on weekends (*P* = 0.001).

There were significant sex differences in recreational ST. For inner-city adolescents, the prevalence of screen device use and total recreational ST was higher among boys compared with girls, with the exception of mobile phone use. A higher proportion of girls than boys reported playing with mobile phones, but girls spent less time using a mobile phone. Overall, the prevalence of ≥2 h/day ST was higher among boys than girls on both weekdays (inner city: 18.9% vs 13.0%, *P* < 0.001; peri-urban: 19.1% vs 12.2%, *P* = 0.005) and weekends (inner city: 75.7% vs 66.2%, *P* < 0.001; peri-urban: 79.3% vs 72.5%, *P* = 0.01).

### ST correlates

Table 3 shows the correlates of adolescents’ recreational ST, adjusted for all variables and BMI category in the model. A significant regional difference was found for weekend ST, with inner-city adolescents being less likely to spend ≥2 h/day recreational ST on weekends than their peri-urban counterparts (odds ratio 0.69, 95% confidence interval 0.57–0.84). On both weekdays and weekend days, ≥2 h/day recreational ST was associated with being male, having a father without a university degree, mothers’ TV time exceeding 2 h, not having family ST rules, eating meals in front of the TV, owning a personal mobile phone, and having a TV in the bedroom. Living with a sibling was associated with an increased risk of excessive ST on weekdays but not on weekends.

**Table 3.** Independent correlates of ≥2 h/day ST in Chinese adolescents based on bivariate and multiple logistic regressions

	Weekdays		Weekends	
	
	Crude OR (95% CI)	Adjusted OR (95% CI)	Crude OR (95% CI)	Adjusted OR (95% CI)
Living in inner city (Shanghai)	1.03 (0.61–1.71)	0.96 (0.74–1.24)	0.78 (0.61–0.99)	**0.69 (0.57–0.84)**
Age (years)	**1.39 (1.21–1.60)**	**1.35 (1.15–1.59)**	**1.40 (1.26–1.57)**	**1.45 (1.28–1.65)**
Being a boy	**1.60 (1.37–1.87)**	**1.71 (1.34–2.16)**	**1.54 (1.27–1.88)**	**1.50 (1.26–1.79)**
Living with a sibling	**1.52 (1.31–1.76)**	**1.77 (1.38–2.28)**	0.84 (0.70–1.00)	0.90 (0.74–1.09)
Father with no university degree	**2.76 (1.84–4.12)**	**2.14 (1.39–3.28)**	**1.54 (1.26–1.89)**	**1.42 (1.11–1.81)**
Mother with no university degree	**2.80 (1.82–4.32)**	**1.76 (1.07–2.90)**	1.31 (0.94–1.82)	0.93 (0.70–1.23)
Father TV time ≥2 h/day	**1.49 (1.25–1.78)**	1.13 (0.88–1.45)	**1.40 (1.18–1.66)**	1.08 (0.90–1.29)
Father computer time ≥2 h/day	0.82 (0.66–1.02)	0.86 (0.66–1.12)	1.02 (0.81–1.28)	1.06 (0.88–1.28)
Mother TV time ≥2 h/day	**1.68 (1.28–2.20)**	**1.44 (1.12–1.85)**	**1.65 (1.34–2.02)**	**1.55 (1.29–1.87)**
Mother computer time ≥2 h/day	0.99 (0.77–1.29)	1.15 (0.88–1.51)	1.09 (0.86–1.37)	1.20 (0.98–1.47)
No ST rules at home	**1.54 (1.17–2.03)**	**1.49 (1.18–1.88)**	**1.38 (1.20–1.57)**	**1.33 (1.12–1.57)**
Family eating meals while watching TV	**2.08 (1.52–2.84)**	**1.31 (1.04–1.66)**	**1.66 (1.31–2.09)**	**1.67 (1.39–2.01)**
Having a mobile phone	1.31 (0.88–1.93)	**1.56 (1.19–2.04)**	1.08 (0.77–1.51)	**1.24 (1.03–1.50)**
Having a TV in the bedroom	**2.44 (1.91–3.12)**	**2.22 (1.75–2.81)**	**1.52 (1.21–1.91)**	**1.35 (1.11–1.64)**
Having a computer in the bedroom	**1.65 (1.31–2.07)**	1.21 (0.94–1.56)	**1.25 (1.11–1.40)**	1.18 (0.97–1.43)
Having a video game console in the bedroom	**2.08 (1.52–2.84)**	**1.70 (1.30–2.23)**	**1.66 (1.31–2.09)**	1.27 (0.99–1.63)

CI, confidence interval; OR, odds ratio; ST, screen time; TV, television.

Bolded numbers indicate significant associations.

Adjusted ORs were calculated adjusted for all other variables and BMI category in the model.

## DISCUSSION

In the present study, we found that, in two eastern Chinese cities with different degrees of urbanization, the majority of adolescents exceeded the recommended ST on weekends, with boys reporting higher total daily ST than girls. These findings are similar to ST estimates among western adolescents.^7^^,^^15^

There were some regional differences in Chinese adolescents’ recreational ST behavior, and these differences varied according to screen device. On weekdays, the proportion of inner-city adolescents engaging in recreational ST was consistently higher than that of peri-urban adolescents, although the prevalence of exceeding the ST recommendation between regions did not differ. On weekends, peri-urban adolescents were heavier users of TV in terms of both prevalence and duration. Inner-city boys spent more time on computers, while a higher proportion of inner-city girls spent their discretionary time playing with mobile phones than did girls from the peri-urban area. Such differences show that, compared to their peri-urban counterparts, inner-city adolescents were more likely to choose other media entertainment over traditional TV viewing. Also, inner-city girls seemed to have the lowest ST among all children. One possible explanation is that Chinese girls, rather than boys, are more likely to be involved in other non-ST activities (either tutoring and/or other extracurricular activities), and such opportunities may be less common in peri-urban families. Additional analysis of the current study found that inner-city girls reported significantly more extracurricular classes per week than inner-city boys (1.34 vs 1.03 classes, *P* < 0.001) and peri-urban boys and girls (0.42, *P* < 0.001, and 0.62, *P* < 0.001, respectively). Furthermore, Cui et al also found that among adolescents aged 13–18 years, girls reported more extracurricular activities than boys (0.5 vs 0.4, *P* < 0.001).^9^ Therefore, special attention should be paid to peri-urban families where limited alternative recreational activities are provided to adolescents.

The most prevalent ST behavior in both regions was television viewing. However, in terms of both prevalence and duration, inner-city boys were the heaviest users of computers among all four subgroups. Studies conducted among Hong Kong children and adolescents^16^^,^^17^ also show that boys and girls differed more with respect to computer playing than TV viewing in terms of both duration and prevalence. Such differences may reflect a shift in young people’s entertainment choices in a more urbanized environment. Indeed, boys of higher socioeconomic status tend to be early adopters of new technology, a trend which has also been observed among adolescent boys in other developing countries.^18^^,^^19^

To date, the research on recreational ST has mainly focused on measures of TV, computer, video, and e-game use.^20^ In a recent study of screen time among Chinese children, TV viewing time was still used as the surrogate of ST.^9^ However, the exponential growth in different types of screen technology suggests that such measures of ST may be underestimating ST across different screen types. Our results show that mobile phone playing did not differ between weekdays and weekends, which demonstrates the pervasive nature of mobile phone usage. Interestingly, the prevalence of mobile phone playing was higher among girls than boys; however, inner-city boys reported spending more time on mobile phones than girls. In contrast, Soederqvist et al^21^ found that Swedish girls were heavier users of mobile phones in terms of both access and duration. However, their study was conducted six years before our own, when mobile phones were mainly used for communication. In our study, measurement of mobile phone usage was focused on mobile phone playing. With recent advance in smartphone technologies, mobile phones have become increasingly multi-functional. Therefore, boys may become heavier mobile-phone users than girls when surfing and playing games become major functions of mobile phones.

In the past decade, China has become the leading consumer market for mobile phones.^22^ According to a 2011 Nielson report, Chinese youth are the leading users of mobile phones for internet access in China, with 70% of 15–24 years old youth reporting that they use their mobile phone to access the internet.^23^ As shown in the current study, playing on computers and mobile phones were more popular ST activities than playing with video game consoles. A possible explanation is that parents may be less likely to interfere, or sometimes even encourage, the use of computers/tablets/smartphones. The multi-functional nature of these devices may obscure parents’ awareness of their potential negative effects on health. Hence, it may be worth communicating the potential harms of excessive use of computers or tablet devices, even when it is for educational purposes.

More than half of adolescents in the present study reported having two or more screen devices in their bedrooms and that there were no rules for their recreational ST. Further, over three-quarters of adolescents’ parents reported high personal ST. On both weekdays and weekends, parents’ role modeling (mothers’ TV time and family eating in front of TV) and fathers’ education levels were correlated with adolescents’ ST. These findings are consistent with other studies conducted in developed countries.^7^^,^^8^ Lack of family rules regarding ST was also related to excessive ST on both weekdays and weekends, which was not found in a previous study.^9^ This suggests that health education should engage parents to be aware of not only the health risks associated with high ST but also how they could positively influence children by enforcing family rules and changing their own behaviors, especially among families of low socioeconomic status.

### Strengths and limitations

To our knowledge, this is the first study with a large random sample to examine adolescents’ ST behaviors in China and to investigate regional differences between adolescents living in inner-city and peri-urban areas. One strength of this study is that ST behaviors were comprehensively measured, particularly including mobile phone playing. However, we did not ask about the type of mobile phone (ie smartphone versus traditional mobile phone) or the primary function of the mobile phone (eg, messaging, social media, or playing games). The study is cross-sectional, and is only based on self-reports of adolescents with possible recall bias. Parents’ screen behaviors were reported by only one of the parents and may be inaccurate and subject to recall bias. The measures of ST behaviors have not been validated against objective measures, but they are comparable to measures used by other studies.^24^^,^^25^ Also, we understand that using 2 h/day as a cut-off point may not necessarily be an appropriate approach for Chinese adolescents, but such a cut-off makes our findings comparable to those of other studies. Finally, the study was conducted in a specific geographic region in China, and findings may not be generalizable to other areas in China.

### Conclusions

The gender and type-of-day differences in recreational ST among urban Chinese adolescents are similar to those reported in other countries. The popularity of computer playing and mobile phone usage among adolescents, especially in those from more urbanized areas, should raise concerns. Parental role modeling and access to screen devices have been consistently associated with adolescents’ ST, including in this study. A clear message that advocates the engagement of parents to create a healthier family environment is warranted. More population-level surveillance and research is needed to monitor the trends of recreational ST behaviors and to better understand the characteristics of those who are at risk. Additionally, further research is required to determine which devices comprise recreational ST, especially mobile phone use, and what functions of mobile phone usage are contributing the most to adolescents’ ST.
